# Long noncoding RNAs: fine-tuners hidden in the cancer signaling network

**DOI:** 10.1038/s41420-021-00678-8

**Published:** 2021-10-11

**Authors:** Shanshan Zhao, Xue Zhang, Shuo Chen, Song Zhang

**Affiliations:** 1 Key Laboratory of Reproductive Dysfunction Diseases and Fertility Remodeling of Liaoning Province, Reproductive Medicine Center, Obstetrics and Gynecology Department, Shengjing Hospital Affiliated to China Medical University, 110022 Shenyang, Liaoning China; 2 Department of Epigenetics, China Medical University, 110122 Shenyang, Liaoning China; 3 Department of Obstetrics and Gynecology, Department of Gynecologic Oncology Research Office, Key Laboratory for Major Obstetric Diseases of Guangdong Province, The Third Affiliated Hospital of Guangzhou Medical University, 510150 Guangzhou, Guangdong China; 4 Department of Thoracic Surgery, The First Affiliated Hospital of China Medical University, 110001 Shenyang, Liaoning China; 5 Department of Environmental and Occupational Health, School of Public Health, China Medical University, 110122 Shenyang, Liaoning China

**Keywords:** Tumour biomarkers, Oncogenesis, Tumour biomarkers

## Abstract

With the development of sequencing technology, a large number of long non-coding RNAs (lncRNAs) have been identified in addition to coding genes. LncRNAs, originally considered as junk RNA, are dysregulated in various types of cancer. Although protein-coding signaling pathways underlie various biological activities, and abnormal signal transduction is a key trigger and indicator for tumorigenesis and cancer progression, lncRNAs are sparking keen interest due to their versatile roles in fine-tuning signaling pathways. We are just beginning to scratch the surface of lncRNAs. Therefore, despite the fact that lncRNAs drive malignant phenotypes from multiple perspectives, in this review, we focus on important signaling pathways modulated by lncRNAs in cancer to demonstrate an up-to-date understanding of this emerging field.

## Facts

- Long noncoding RNAs are >200 nt in length and have almost no protein-encoding function, but they can exert regulatory effects on transcriptional, post-transcriptional, and post-translational modifications of coding genes.
- Signaling pathways are a series of enzymatic reactions in response to intracellular and extracellular stimuli and underlie almost every disease, including cancer.
- LncRNAs participate in tumorigenesis and cancer progression by fine-tuning various cancer signaling pathways.

## Open questions

- Whether disparate signaling pathways regulated by an individual lncRNA converge on similar cancer hallmarks?
- What are the mechanisms through which the crosstalk among diverse signaling pathways is established?
- How can we target lncRNAs to fine-tune cancer signaling pathways?

## Introduction

Recently, many noncoding RNAs have been discovered using high-throughput sequencing technology. Although they do not encode proteins, they have powerful functions, participating in transcriptional, post-transcriptional, and post-translational regulation of coding genes. Long noncoding RNAs (lncRNAs) are >200 nt in length and have almost no protein-encoding function [1]. LncRNAs are further divided into different subclasses, including intergenic transcripts, enhancer RNAs, and sense or antisense transcripts, according to their locations or relationship with protein-coding protein [2]. LncRNAs perform various functions at transcriptional and translational levels, acting in *cis* or *trans* [3]. They play important roles in many biological activities, such as genomic imprinting and X-chromosome inactivation, and participate in cellular behaviors, such as proliferation, differentiation, and survival [4, 5]. They are also implicated in cancer hallmarks and are involved in tumorigenesis and cancer progression [6, 7].

Protein is the executor of biological function. Protein-coding signaling pathways underlie almost every biological process, especially carcinogenesis and progression. Signaling pathways are a series of enzymatic reactions in response to intracellular and extracellular stimuli. They participate in the transmission of cellular signals and are important for biological reactions and gene expression. Signaling pathways are extremely complex, and many signaling molecules are involved in cascade reactions, which are often regulated, directly or indirectly, by various types of molecules [8]. Emerging evidence shows that abnormal signal transduction is a key trigger and indicator for tumorigenesis and cancer progression and that lncRNAs, as versatile regulators, can shape the malignant phenotypes by activating or suppressing different signaling pathways [9, 10]. In this review, we selectively discuss those signaling pathways that are fine-tuned by lncRNAs and well studied mechanistically in tumors, such as Wnt/β-catenin, Hippo, Notch, and nuclear factor (NF)-κB pathway. While we did our best to cover this topic, some additional publications may also help get the full picture [10–12].

## The Wnt/β-catenin signaling pathway

Wnt/β-catenin signaling pathway is the canonical pathway of the Wnt signaling cascade. Its dysregulation is implicated in tumorigenesis, including proliferation, invasion, metastasis, and apoptosis, and has been the focus of cancer research [13–17]. The Wnt/β-catenin signaling pathway starts from Wnt ligands binding to a receptor complex, comprising Frizzled (Fz), and its co-receptor, low-density lipoprotein receptor-related protein 5/6. When a ligand attaches, the receptor is activated and the protein disheveled homolog (DVL) is phosphorylated, resulting in deactivation of the destruction complex, which comprises AXIN, adenomatous polyposis coli, glycogen synthase kinase 3β (GSK3β), and casein kinase 1α (CK1α). Non-phosphorylated β-catenin accumulates in the cytoplasm and migrates to the nucleus, where it interacts with T cell-specific factor (TCF)/lymphoid enhancer-binding factor transcription factors and triggers the expression of downstream genes, such as those encoding c-Myc, cyclin D1, and CDKN1A [16, 18, 19]. Under the inactivation of Wnt signaling, GSK3β and CK1α within the degradation complex phosphorylate β-catenin, which then undergoes ubiquitin-mediated proteolysis [20, 21].

LncRNAs play a tumor-suppressive or tumor-promoting role via interacting with key factors in the Wnt pathway. For instance, in lung adenocarcinoma, lncRNA LINC00673 enhances the interaction between DDX3 and CK1ε to phosphorylate DVL, thereby activating the Wnt /β-catenin signaling pathway and promoting cancer progression [22]. LncRNAs can inhibit β-catenin phosphorylation by interacting with key enzymes in the destruction complex, thus activating the Wnt/β-catenin signaling pathway. The binding of LncRNAs CYTOR and SLCO4A1-AS1 to β-catenin impedes CK1- and GSKβ-mediated phosphorylation of β-catenin, respectively, leading to β-catenin accumulation and translocation to the nucleus. Activation of the Wnt/β-catenin signaling pathway facilitates colorectal cancer growth and metastasis [23, 24]. As the central protein of the Wnt/β-catenin signaling pathway, the function of β-catenin is closely related to its stability and interacting proteins. Linc01354 contributes to the stability of β-catenin mRNA by interacting with hnRNP-D and the activated Wnt/β-catenin signaling pathway aggravates colorectal cancer [25]. Linc00210 interacts with CTNNBIP1 and blocks its inhibitory role in Wnt/β-catenin activation, playing a tumor-promoting role in liver tumorigenesis [26]. LncRNAs can also inhibit Wnt/β-catenin signaling. Linc01197 impedes the β-catenin–TCF4 interaction by occupying the β-catenin-binding site, thus restraining Wnt/β-catenin signaling and inhibiting pancreatic adenocarcinoma progression [27]. Besides, lncRNAs affect downstream gene expression by regulating transcription factors in Wnt/β-catenin signaling. LincTCF7 regulates TCF7 expression by recruiting the SWI/SNF complex to its promoter, causing activation of Wnt signaling, and thus promoting liver cancer stem cell self-renewal [28].

**Fig. 1:**
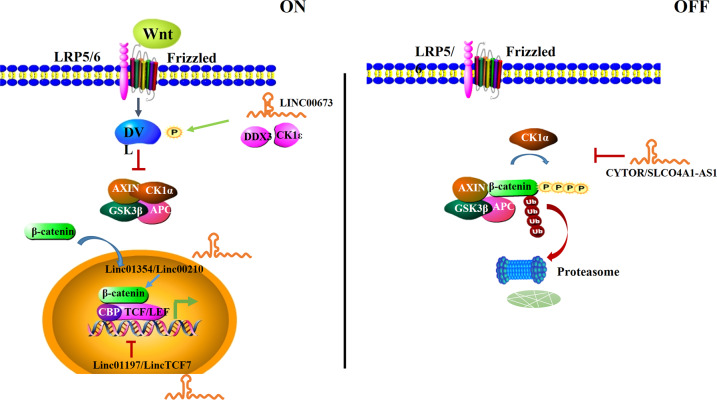
Schematic of the canonical Wnt/β-catenin signaling pathway and the interactions between lncRNAs and the pathway. “Wnt ON”: When Wnt ligands bind to a receptor complex, including Frizzled (Fz), and its co-receptor, LRP5/6, the receptor is activated and DVL phosphorylation occurs. This leads to the deactivation of the destruction complex, which is composed of AXIN, APC, GSK3b, and CK1a. Subsequently, stabilized β-catenin migrates to the nucleus to interact with TCF/LEF and trigger the downstream gene expression. “Wnt OFF”: In the absence of Wnt ligand, GSK3β and CK1α phosphorylate β-catenin, resulting in ubiquitin-mediated proteolysis of β-catenin.

Collectively, lncRNAs can regulate multiple molecules of the Wnt/β-catenin signaling pathway, ultimately facilitating malignant phenotypes (Fig. 1).

## The Hippo signaling pathway

Hippo signaling is an evolutionarily highly conserved pathway that regulates multiple biological processes including cell proliferation and differentiation. Disruption of the Hippo pathway may trigger tumorigenesis. However, mutations in Hippo pathway genes are not common. Molecular events that interfere with the Hippo pathway might be an important mechanism in cancer initiation [29]. The Hippo pathway is mainly regulated by different cell- and tissue-level characteristics, such as cell-to-cell adhesion, basement membrane adhesion, planar cell polarity, and mechanical forces perceived by the actin cytoskeleton [30]. After receiving the upstream signals, the core kinase combination, including LATS1, LATS2, MST1, and MST2, and the SAV1 and MOB1 adaptor proteins, are activated. They cooperate to phosphorylate transcriptional coactivators yes associated protein (YAP) and TAZ [30] and promote cytoplasmic retention of YAP and TAZ by creating a binding site on 14-3-3 proteins [31]. Additionally, re-phosphorylation of YAP/TAZ results in proteasomal degradation [32]. When dephosphorylation of YAP and TAZ occurs, they translocate to the nucleus and promote tissue growth by interacting with transcription factors, such as TEADs and SMADs [33].

As mentioned above, molecular events might play an important role in regulating the Hippo pathway. Many lncRNAs are involved in regulating this pathway. LncRNAs can interact with the core kinase, thus affecting the downstream cascade. LncRNA MIR100HG binds to the histone methylation regulator EZH2, acting as a scaffold that epigenetically silences LATS1/2. MIR100HG plays an oncogenic role in osteosarcoma through inactivating the Hippo pathway [34]. LncRNA MAYA participates in the ROR1/HER3-LLGL2-MAYA-NSUN6 signaling axis, mediating the NSUN6-dependent methylation of MST1, which abolishes kinase activity and activates the HIPPO pathway. This might provide a new therapeutic direction for bone metastasis [35]. LncRNAs can also target the important downstream executor, YAP, in the Hippo pathway. LncRNA RP11‑323N12.5 is upregulated in gastric cancer and exerts its tumor-promoting and immunosuppression role by activating the Hippo pathway. RP11‑323N12.5 promotes the transcription of YAP1 by binding to c-MYC on the YAP1 promoter. In turn, YAP1/TAZ/TEADs can also regulate RP11-323N12.5 transcription by forming a positive feedback loop [36]. Moreover, LncRNAs can affect the localization of YAP. LncRNA UCA1, which is overexpressed in pancreatic cancer, can inhibit the phosphorylation of YAP by forming shielding composites with MOB1, LATS1, and YAP. Consequently, YAP accumulates in the nucleus and activates the downstream genes of the Hippo signaling pathway [37].

**Fig. 2:**
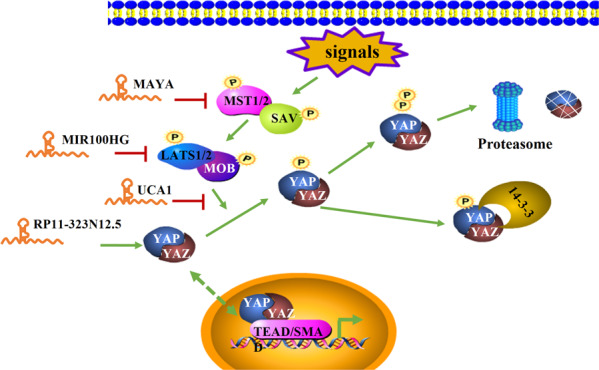
Schematic of the Hippo signaling pathway and the interaction between lncRNAs and the pathway. After receiving the upstream signals, the core kinase combination, including LATS1, LATS2, MST1, MST2, SAV1, and MOB1, are activated and cooperate to phosphorylate YAP and TAZ. Phosphorylated YAP/TAZ underdo retention by 14-3-3 proteins or proteasomal degradation. When dephosphorylation of YAP and TAZ occurs, they translocate to the nucleus and interact with TEADs and SMADs to promote downstream gene transcription.

Thus, lncRNAs modulate the Hippo signaling pathway in diverse manners, eventually leading to the activation or suppression of downstream cancer-related genes (Fig. 2).

## The Notch signaling pathway

Notch signaling pathway is involved in various biological processes, including cell differentiation, proliferation, and apoptosis. Notch signaling also plays pleiotropic roles in carcinogenesis and tumor progression. Four receptors (Notch 1, 2, 3, and 4) and five ligands (DLL 1, 3, and 4; JAG 1 and 2) have been identified in Notch signaling [38]. The receptor–ligand interactions trigger sequential Notch receptor cleavage by the ADAM family of metalloproteases and γ-secretase, leading to the release of the Notch intracellular domain (NICD). NICD translocates to the nucleus and binds to the CBF-1/Su(H)/LAG1 transcription factor, displacing co-repressors, and recruiting co-activators such as Mastermind-like (MAML), thus inducing Notch target gene transcription [39]. Depending on the strength and dynamics of juxtacrine signaling, Notch pathway activates different programs that determine cell fate [40]. Notch signaling is involved in the regulation of tumor-related genes, such as those encoding MYC, cyclin-D1, or p21 [41].

LINC01152 is overexpressed in glioblastoma multiforme, functioning as an oncogene. It activates the Notch signaling pathway by upregulating the expression of transcriptional co-activator 2 (MAML2). LINC01152 serves as a competing endogenous RNA via sponging microRNA miR-466. Moreover, LINC01152 can prevent MAML2 from shearing via recruiting SRSF1, thus maintaining the level of MAML2. Besides, MAML2 could, in turn, promote the expression of LINC01152 via regulating the Notch pathway [42]. LncRNA CEBPA-AS1 acts as a tumor suppressor in osteosarcoma by inhibiting Notch signaling pathway activity. LncRNA CEBPA-AS1 binds to miR-10b-5p and regulates the activity of NCOR2, which is a nuclear receptor co-repressor. Taken together, CEBPA-AS1 overexpression inhibits osteosarcoma progression by suppressing the Notch signaling pathway via upregulating NCOR2 expression [43].

**Fig. 3:**
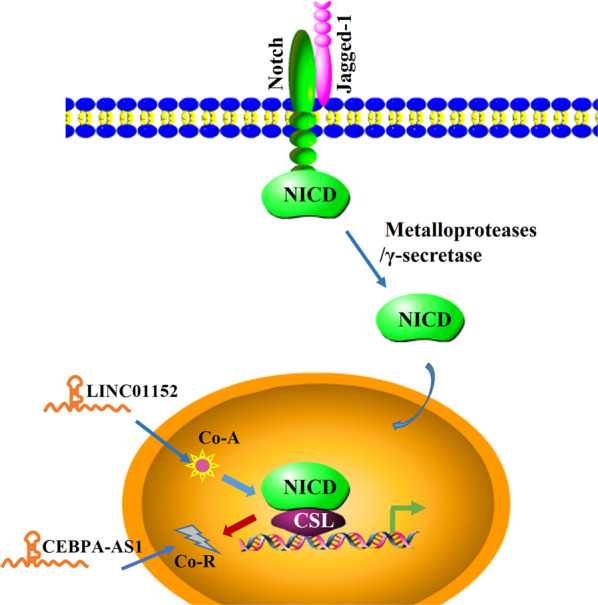
Schematic of the Notch signaling pathway and the interactions between lncRNAs and the pathway. After Jagged-1 ligand binding to the Notch receptor, the Notch receptor is cleaved by ADAM family of metalloproteases and γ-secretase, leading to the release of NICD. NICD translocates to the nucleus and binds to CBF-1/Su(H)/LAG1 (CSL) transcription factor, displacing co-repressors (Co-Rs), and recruiting co-activators (Co-As), to induce target gene transcription.

Although the Notch signaling pathway has a relatively simple framework, it plays an important role in cell proliferation, differentiation, and apoptosis (Fig. 3). As mentioned above, lncRNAs are involved in various steps of this pathway. However, further effort is required to determine the details.

## The NF-κB signaling pathway

Since the NF-κB subunits were recognized >20 years ago as being homologous to the viral oncogene v-Rel, the role of these proteins in tumorigenesis has been accepted. However, early research on NF-κB mainly focused on the regulation of the immune response. With the deepening of the research on NF-κB, the role of the inflammatory response in tumor development gradually became prominent [44, 45]. NF-κB activation enhances the hallmarks of cancer (such as proliferation, invasion, metastasis, angiogenesis, and prevention of apoptosis), thus supporting tumorigenesis [46].

In mammals, NF-κB complexes comprise five proteins, including RelA (p65), RelB, c-Rel, p50 (p105 precursor), and p52 (p100 precursor), which all share an N-terminal Rel homology domain that is required for dimerization, DNA binding, and inhibitory IκB protein interaction [47]. There are two distinct modes: canonical (activated by tumor necrosis factor (TNF), interleukin-1 (IL-1), and Toll-like receptor ligands) and non-canonical (activated by TNF superfamily receptors, B cell activating factor receptor, and receptor-activated NF-κB ligand) [48]. In the canonical pathway, generally, NF-κB is inhibited in an inactive state in the cytoplasm by a family of inhibitor of NF-κB (IκB) proteins, which consists of IκBα, IκBβ, and IκBε [49]. Following exposure to an inducing stimulus, IκB is phosphorylated by activated IKK2, part of the IκB kinase (IKK) complex, which consists of two catalytic subunits, IKKα (IKK1) and IKKβ (IKK2), and the non-catalytic regulatory protein NEMO or IKKγ. The phosphorylated IκB then undergoes ubiquitination and proteasome-mediated degradation, together with NF-κB nuclear localization [50]. In the non-canonical pathway, IKKα is phosphorylated by NF-κB-inducing kinase following receptor ligation. Activated IKKα induces p100 phosphorylation, which undergoes partial proteolysis to p52 [51]. Various NF-κB dimers released by either the canonical or non-canonical pathways translocate to the nucleus and interact with transcription factors and cofactors (such as TBP, TFIIB, p300, and CBP) to trigger target gene transcription [52].

As an important signaling pathway, NF-κB signaling is modulated by multiple regulatory mechanisms. Increasing evidence shows that lncRNAs are involved in the regulation of the NF-κB signaling pathway. LncRNAs can directly interact with functional signaling proteins involved in NF-κB signaling, serving as modulators. LncRNA NKILA inhibits IKK-induced IκB phosphorylation by masking the phosphorylation motifs of IκB and impeding NF-κB activation. NKILA prevents breast cancer metastasis by inhibiting over-activation of the NF-κB pathway [53]. In gastric cancer, lncRNA KRT19P3 could suppress cancer cell proliferation and metastasis by regulating the expression of COPS7A. COPS7A can promote deubiquitinylation of IκBα, which inactivates the NF-κB signaling pathway [54]. In addition, lncRNA SChLAP1 interacts with HNRNPL, forming a complex, which stabilizes ACTN4 by preventing its proteasomal degradation. The stabilization of ACTN4 increases the p65 subunit nuclear localization and activates NF-κB signaling. Therefore, SChLAP1 acts as a growth-promoting lncRNA in glioblastoma [55].

**Fig. 4:**
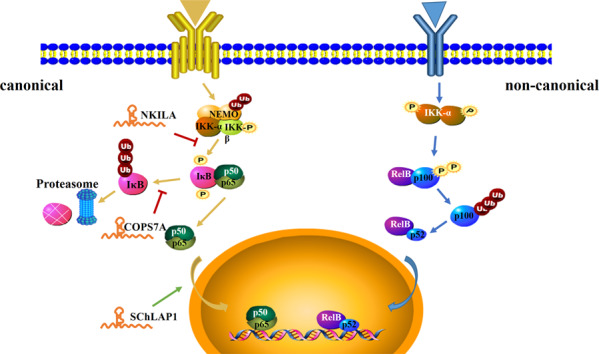
Schematic of the NF-κB signaling pathway and the interactions between lncRNAs and the pathway. NF-κB consists of five subunits, RelA (p65), RelB, c-Rel, p50 (p105 precursor), and p52 (p100 precursor). In the canonical pathway: followed by exposure to an inducing stimulus, IκB is phosphorylated by activated IKKβ. The phosphorylated IκB then undergoes ubiquitination and proteasome-mediated degradation along with NF-κB nuclear localization. In the non-canonical pathway: IκB kinase is phosphorylated by NF-κB-inducing kinase following receptor ligation. Activated IKKα induces p100 phosphorylation, which undergoes partial proteolysis to p52 and translocates to the nucleus.

LncRNAs are emerging as important modulators in the management of NF-κB signaling, gradually unraveling the mystery of cancer development and progression (Fig. 4).

## The Hedgehog (HH) signaling pathway

The HH signaling pathway is essential for many fundamental processes, including embryogenesis, stem cell maintenance, and tissue regeneration [56]. Some studies indicated that HH signaling plays a broader role in carcinogenesis, such as in breast cancer, gastric cancer, and prostate cancer [56–59]. Here we present a brief overview of the HH signaling pathway. In mammals, there are three HH ligands (SHH, DHH, and IHH), one 12-pass transmembrane receptor PTCH1, one G protein-coupled receptor (GPCR)-like seven-pass transmembrane protein, SMO, and three transcription factors (GLI1, GLI2, and GLI3) in the HH signaling cascade. All components of the HH signal pathway were discovered in the primary cilia [60]. In the absence of HH ligands, PTCH1 suppresses the accumulation of SMO, thus repressing its activity [61]. Transcription factors GLI2 and GLI3 are trapped in the cytoplasm by SUFU and are consequently phosphorylated by protein kinase A, CK1, and GSK3β. Phosphorylated GLI2 and GLI3 are degraded by the proteasome into repressor forms (GLI2R and GLI3R) [62]. GLIR binds to the promoters of HH signaling target genes and silences their transcription [63]. Once HH binds to PTCH1, SMO inhibition is relieved. Activated SMO relieves the suppression of GLI2 and GLI3 by SUFU. The GLIs evade phosphorylation and the activated forms (GLI2A and GLI3A) translocate to the nucleus to activate HH target gene expression [64, 65].

**Fig. 5:**
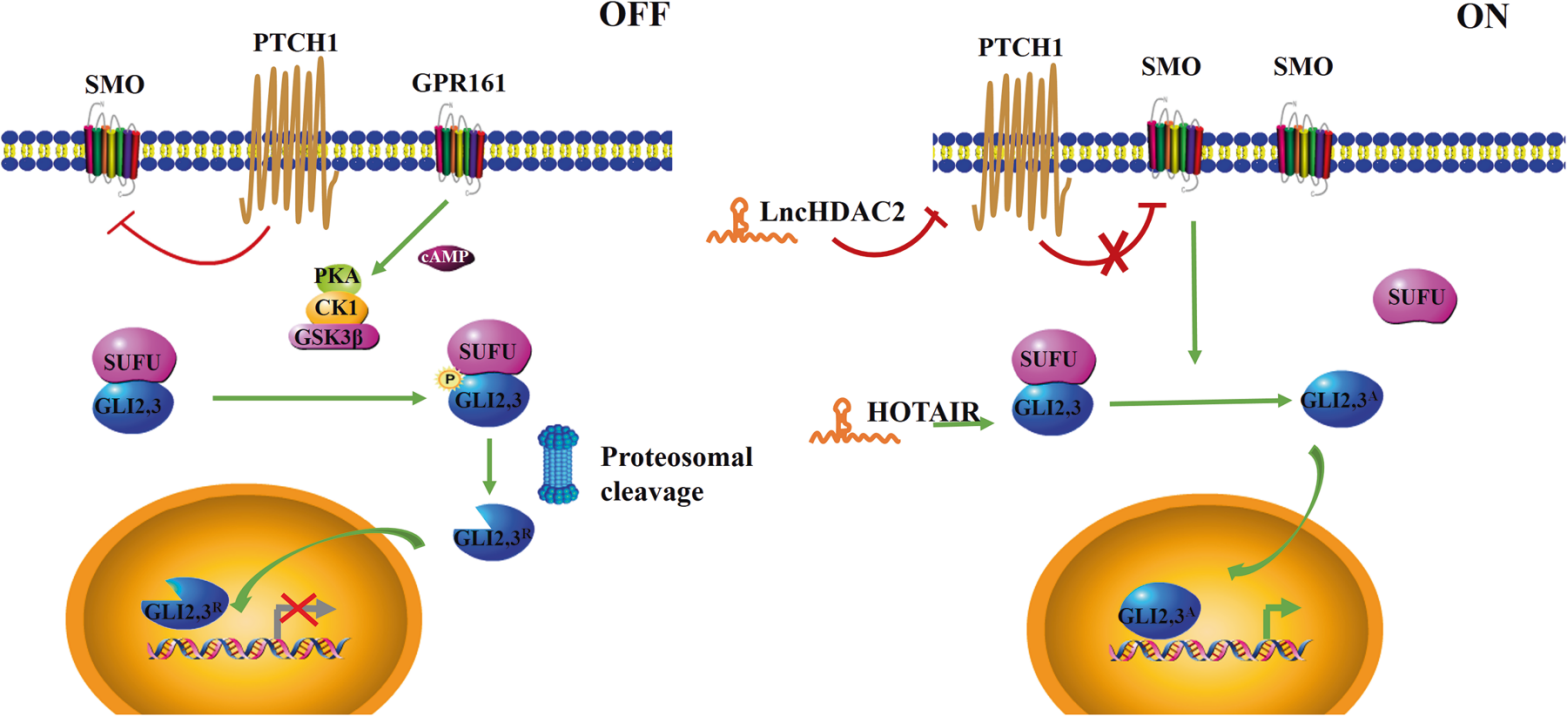
Schematic of the Hedgehog signaling pathway and the interactions between lncRNAs and the pathway. “Hedgehog OFF”: In the absence of Hedgehog ligands, PTCH1 suppresses the accumulation of SMO, thus repressing its activity. GLI2 and GLI3 are trapped in the cytoplasm by SUFU and consequently phosphorylated by PKA, CK1, and GSK3β. Phosphorylated GLI2 and GLI3 are degraded by the proteasome into repressor forms (GLI2^R^ and GLI3^R^), which inhibit downstream gene expression. “Hedgehog ON”: Once Hedgehog binds to PTCH1, SMO inhibition is relieved. Activated SMO relieves the suppression of GLI2 and GLI3 by SUFU. The GLIs evade phosphorylation and the activated forms (GLI2^A^ and GLI3^A^) translocate to the nucleus to activate Hedgehog target gene expression.

LncRNAs, as small-molecule regulators, may be involved in modulating the HH signaling pathway at different levels and might be related to tumorigenesis and development. LncRNA HOTAIR expression is dysregulated in various cancers and affects cancer development through multiple mechanisms [66]. In renal cell carcinoma, highly expressed HOTAIR interacts with the androgen receptor (AR) and cooperatively promotes GLI2 transcription by binding to its promoter. Increased GLI2 activates the HH signaling pathway and promotes the expression of its downstream genes, which are associated with tumor angiogenesis and cancer stemness [67]. LncHDAC2 is highly expressed in liver cancer and drives cancer stem cell self-renewal and tumor propagation. Mechanistically, lncHDAC2 inhibits PTCH1 expression via recruiting the NuRD complex to its promoter, resulting in activation of HH signaling [68] (Fig. 5). Further study of the regulation of HH signaling pathway by lncRNAs in cancer will clarify the regulatory network between them.

## The transforming growth factor (TGF)-β signaling pathway

The TGF-β signaling pathway consists of TGF-β ligands, receptors, and downstream signaling molecules. The pathway participates in organismic homeostasis, controlling cell proliferation, motility, and differentiation [69, 70]. In normal tissues, the TGF-β pathway maintains homeostasis, and once tumorigenesis has progressed, TGF-β is involved in promoting tumor malignancy [71–73]. The cascade initiates from the binding of TGF-β ligands to TGF-β type II receptors (TGFBR2), subsequently recruiting and phosphorylating TGF-β type I (TGFBR1). In the canonical pathway, phosphorylated TGFBR1, in turn, phosphorylates the signaling proteins SMAD2 and SMAD3. Following phosphorylation, they form a complex with SMAD4. Subsequently, the complex translocates to the nucleus and regulates downstream gene expression, together with cofactors and transcription factors [74]. Meanwhile, another class of SMAD proteins functions as inhibitors of the TGF-β signaling pathway, called I-SMADs, comprising SMAD6 and SMAD7. They attach directly to TGFBR1, thereby interrupting subsequent phosphorylation of SMADs or hindering the formation of the SMAD2,3/SMAD-4 complex [75]. In addition to SMAD proteins, TGF-β receptors also induce other signal transducers, including mitogen-activated protein kinase, phosphoinositide-3 kinase (PI3K)/AKT, Janus-activated kinase (JAK)/signal transducer and activator of transcription factor (STAT), and c-Jun N-terminal kinase/p38 in non-canonical pathway [76, 77].

**Fig. 6:**
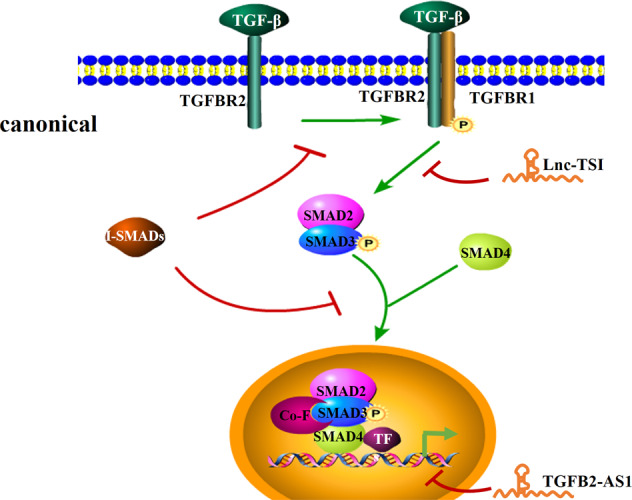
Schematic of the TGF-β signaling pathway and the interaction between lncRNAs and the pathway. TGF-β ligands bind to TGF-β type II receptors (TGFBR2), subsequently recruiting and phosphorylating TGF-β type I (TGFBR1). The phosphorylated TGFBR1 phosphorylates the signaling proteins SMAD2 and SMAD3. Following phosphorylation, they form a complex with SMAD4. The complex translocates to the nucleus and regulates downstream gene expression. Inhibitors, termed I-SMADs, attach directly to TGFBR1 and intercept the phosphorylation of SMADs or hinder the formation of the SMAD2, 3/SMAD-4 complex.

Many studies have confirmed that lncRNAs are involved in the regulation of the TGF-β signaling pathway, thus affecting tumorigenesis and cancer progression. Lnc-TSI exerts a tumor-suppressor role in clear cell renal cell carcinoma by inhibiting metastasis. Lnc-TSI impedes the phosphorylation of SMAD3 by blocking the interaction between SMAD3 and TGFBR1 at the MH2 domain. Consequently, the TGF-β/SMADs signaling pathway is inhibited, which suppresses epithelial–mesenchymal transition [78]. LncRNAs can also regulate the TGF-β signaling pathway by modulating its co-factors. LncRNA TGFB2-AS1 assists repressive histone H3K27me3 modifications at the promoters of TGF-β-target genes by binding to the EED adaptor of the Polycomb repressor complex 2 (PRC2) via the 3’ terminal region of TGFB2-AS1. Thus, TGFB2-AS1 exerts an inhibitory role on TGF-β signaling downstream genes [79]. Additionally, lncRNA MEG3 regulates the activity of TGF-β genes by forming an RNA–DNA triplex structure through GA-rich binding sites. The RNA–DNA triplex might be a general characteristic whereby lncRNAs recognize their target genes [80] (Fig. 6).

Although a large number of lncRNAs have been reported to be related to TGF-β signaling pathway, the exact mechanisms of their mutual interactions are mostly unknown. Further research into the lncRNA–TGF-β signaling network will expand our understanding of cancer pathogenesis and provide novel insight into therapeutic strategies.

## Other signaling pathways

In addition to the signaling pathways mentioned above, lncRNAs also play regulatory roles in other signaling pathways during tumorigenesis. One of these is the JAK-STAT signaling pathway, which is necessary for homeostasis and development, mainly mediating inflammation and immunity [81]. Disturbance of the JAK-STAT signaling pathway might be involved in the cancer hallmarks, such as evasion of immune surveillance and promoting or suppressing tumor progression. Activation of the pathway is initiated from the interaction of extracellular cytokines and other ligands with transmembrane receptors, and thus receptor-bound JAKs are activated. Subsequently, JAK mediates the phosphorylation STATs, which form homodimers or heterodimers and transfer into the nucleus to trigger downstream gene expression [82, 83]. LncRNA LINC00669 facilitates the malignancy of nasopharyngeal carcinoma by competitively binding to suppressor SOCS1 of the JAK/STAT signaling pathway. In this way, LINC00669 protects STAT1 from ubiquitination modification and stabilizes it. STAT1 translocates to the nucleus and promotes the transcription of target genes [84]. Meanwhile, tumor-suppressive lncRNAs, such as RP11-468E2.5, suppress the JAK/STAT signaling pathway by targeting STAT5 and STAT6, thereby inhibiting colorectal cancer cell proliferation and promoting apoptosis [85].

Another potent pathway is the PI3K/AKT signaling pathway, which consists of two main proteins, PI3K and AKT. Activated by growth factors through GPCRs or receptor tyrosine kinase receptors, PI3K activates AKT and downstream effectors through the lipid second messenger phosphatidylinsoitol-3,4,5-trisphosphate. The pathway is important for cell growth and differentiation. Disturbance of the PI3K/AKT pathway might be a critical event in the process of tumor development [86]. It has been demonstrated that the PI3K pathway is one of the most frequently activated pathways in human cancer [87]. LncRNAs serve as indispensable regulators in this signaling cascade. Overexpression of lncRNA DANCR correlates with poor prognosis and low survival rate in triple-negative breast cancer. Mechanically, DANCR interacts with RXRA and increases its phosphorylation via GSK3β, which alleviates the inhibition of PIK3CA transcription and subsequently leads to activation of the PI3K/AKT signaling pathway and tumorigenesis [88]. LncRNAs can indirectly regulate the PI3K signaling pathway by regulating growth factor ligand activity of the cascade. LncRNA PART1, which serves as a tumor suppressor in gastric cancer, epigenetically silences PDGFB (encoding platelet-derived growth factor B) via PLZF-mediated recruitment of EZH2, thus inhibiting PI3K/Akt signaling activation [89].

## LncRNAs regulate signaling pathways of cancer

Deregulation of protein-coding signaling pathways is fundamental to tumorigenesis and cancer progression. However, lncRNAs, hidden in the signaling pathways, cannot be neglected for their fine-tuning functions. Notably, one signaling pathway can be fine-tuned by multiple lncRNAs. As mentioned previously, Linc01354, Linc00210, Linc01197, LincTCF7, and LINC00673 participate in activating or suppressing the Wnt/β-catenin signaling pathway. In another example, lncRNAs, as upstream mediators, have a dual function in the STAT3 signaling pathway in different cancers. NEAT1, SNHG3, and H19 induce STAT3 expression, while MEG3, PTCSC3, and NKILA inhibit STAT3 expression [90]. Not surprisingly, one lncRNA may also regulate several signaling pathways. For example, MALAT1, acting as an oncogene, can activate the Wnt/β-catenin signaling pathway through upregulation of β-catenin as well as downregulation of GSK3β [91]. Meanwhile, MALAT1 increases JAG1 (Jagged1) expression through inhibiting miR-124 and thus activates the Notch signaling pathway [92]. In addition to independently regulating different pathways, lncRNAs can connect different signaling pathways. In breast cancer, lncRNA BCAR4 connects the Hippo and HH signaling pathways. The downstream effector YAP of the Hippo pathway promotes BCAR4 expression, which activates HH signaling to facilitate the transcription of glycolysis activators HK2 and PFKFB3, and thus reprograms glucose metabolism [93]. Table 1 provides a brief overview of lncRNAs regulating the signaling pathways discussed above.

**Table 1:** LncRNAs in the regulation of the cancer signaling pathways.

Pathway	LncRNA	Mode of action	Cancer	Reference
Wnt/β-catenin	Linc00673	Activator	Lung adenocarcinoma	[22]
	CYTOR	Activator	Colon cancer	[23]
	SLCO4A1-AS1	Activator	Colorectal cancer	[24]
	Linc01354	Activator	Colorectal cancer	[25]
	Linc00210	Activator	Liver cancer	[26]
	lncTCF7	Activator	Liver cancer	[28]
	CILA1	Activator	Tongue squamous cell carcinoma	[94]
	CCAT1	Activator	Breast cancer	[95]
	CCAL	Activator	Colorectal cancer	[96]
	NEAT1	Activator	Colorectal cancer	[97, 98]
	Linc01197	Inhibitor	Pancreatic adenocarcinoma	[27]
	Linc01391	Inhibitor	Liver cancer	[99]
	LncRNA-NEF	Inhibitor	Liver cancer	[100]
	OTUD6B-AS1	Inhibitor	Clear cell renal cell	[101]
Hippo signaling pathway	MAYA	Activator	Breast cancer	[35]
	RP11-323N12.5	Activator	Gastric cancer	[36]
	UCA1	Activator	Pancreatic cancer	[37]
	B4GALT1-AS1	Activator	Colon cancer	[102]
	SUNO1	Activator	Colon cancer	[103]
	MIR100HG	Inhibitor	Osteosarcoma	[34]
	SNHG15	Inhibitor	Thyroid carcinoma	[104]
	Linc01314	Inhibitor	Hepatoblastoma	[105]
Notch signaling pathway	Linc01152	Activator	Glioblastoma	[42]
	NALT	Activator	Lymphoblastic leukemia	[106]
	SNHG12	Activator	Osteosarcoma	[107]
	FOXD2-AS1	Activator	Colorectal cancer	[108]
	CEBPA-AS1	Inhibitor	Osteosarcoma	[43]
	MEG3	Inhibitor	Endometrial carcinoma	[109]
NF-κB signaling pathway	SChLAP1	Activator	Glioblastoma	[55]
	PLACT1	Activator	Pancreatic cancer	[110]
	H19	Activator	Melanoma	[111]
	NKILA	Inhibitor	Breast cancer, non-small cell lung cancer	[53, 112]
	KRT19P3	Inhibitor	Gastric cancer	[54]
	miR503HG	Inhibitor	Hepatocellular carcinoma	[113]
Hedgehog signaling pathway	HOTAIR	Activator	Renal cell carcinoma	[67]
	LncHDAC2	Activator	Liver cancer	[68]
	lncRNA-Hh	Activator	Breast cancer	[114]
	LincPINT	Activator	Laryngeal carcinoma	[115]
	ASAP1-IT1	Activator	Cholangiocarcinoma	[116]
	HCG18	Activator	Nasopharyngeal carcinoma	[117]
	EGOT	Inhibitor	Breast cancer	[118]
TGF-β signaling pathway	ELIT-1	Activator	Lung adenocarcinoma/gastric cancer	[119]
	SNHG6	Activator	Colorectal cancer	[120]
	CASC9	Activator	Colorectal cancer	[121]
	ANRIL	Activator	Prostate cancer	[122]
	Linc00941	Activator	Colorectal cancer	[123]
	lnc-TSI	Inhibitor	Clear cell renal cell carcinoma	[78]
	TGFB2-AS1	Inhibitor	Various cancer types	[79]
	CASC2	Inhibitor	Breast cancer	[124]
JAK-STAT signaling pathway	LINC00669	Activator	Nasopharyngeal cancer	[84]
	LINC01116	Activator	Osteosarcoma	[125]
	RP11-468E2.5	Inhibitor	Colorectal cancer	[85]
	HAND2-AS1	Inhibitor	Liver cancer	[126]
PI3K/AKT signaling pathway	DANCR	Activator	Breast cancer	[88]
	FOXD1-AS1	Activator	Gastric cancer	[127]
	PTTG3P	Activator	Liver cancer	[128]
	UCA1	Activator	Osteosarcoma	[129]
	PART1	Inhibitor	Gastric cancer	[89]
	ST3Gal6-AS1	Inhibitor	Colorectal cancer	[130]

## Conclusion

The regulation of signaling pathways by lncRNAs is complex and diverse. Studies have confirmed the importance of lncRNAs in the regulatory network, functioning to make the cascades operate more smoothly or with greater flexibility. LncRNAs may interact directly or indirectly with signaling molecules to regulate signaling pathways. Despite the support of advanced technologies, such as RNA precipitation, RNA immunoprecipitation, crosslinking immunoprecipitation, and RNA interference or CRISPR (clustered regularly interspaced short palindromic repeats) technologies, the complexity of signaling pathways makes research on the exact interaction mechanism between lncRNAs and signaling molecules a challenge. With the further development of experimental techniques, a deeper understanding of lncRNA-mediated signaling transduction will be gained.

Although numerous studies have shown that lncRNAs play significant roles in the regulation of signal networks and even in the development of tumors, the human body is a complex system, and no molecule can achieve the effect of affecting the whole body by one action; therefore, it would make sense to investigate intermolecular synergy. However, in the cascades of signaling pathways, a “butterfly effect” might also be a feature. In this complex network, many fields remain unexplored. Thus, further effort on the influence of lncRNAs on signaling pathways and even the whole biological process will shed light on the mystery of cancer. However, even our current limited knowledge has provided a novel and promising avenue for the fast-emerging field.

## Data Availability

Data sharing is not applicable to this article as no datasets were generated or analyzed during the current study.
